# Histopathological Findings Related to ORISE™ Injectable Submucosa Lifting Agent Used in the Endoscopic Mucosal Resection of Bowel Neoplasms: A Review of Three Cases

**DOI:** 10.1155/2020/6918093

**Published:** 2020-01-29

**Authors:** Patricia N. Ibarra-Arzamendia, Mark G. Hanly

**Affiliations:** ^1^Department of Pathology, Hospital Central de IPS, Asuncion 1741, Paraguay; ^2^Department of Pathology, Baptist MD Anderson Cancer Center, Jacksonville FL 31522, USA

## Abstract

The use of nonsaline injectable lifting agents is now routine in the performance of endoscopic mucosal resection of bowel neoplasms (EMR). These agents are used to elevate the mucosa from the muscularis propria and permit more a complete resection of the lesion while mitigating risk of possible thermal injury to the bowel wall and thus preventing perforation. After injection, these new agents, which are replacing normal saline, often remain present in the tissues for some time following the procedure and may be identified in the resection specimens where they may mimic a number of other conditions such as mucin pools, lymphangiomas, granulomatous inflammation, and amyloid deposition. We describe the histological findings associated with the use of nonsaline injectable mucosal lifting agents. Awareness of these agents and their associated artefact may reduce misdiagnosis or the use of unnecessary ancillary studies and highlights the importance of proving relevant clinical information on submission of specimens for pathological examination.

## 1. Introduction

Endoscopic mucosal resection of bowel lesions is a relatively new procedure increasingly utilized in the removal of large, flat sessile polyps [1]. Successful EMR is dependent on expanding the submucosal space to create adequate elevation of the surface neoplastic mucosa and thus aid in the complete resection of the lesion while minimising risk of thermal or mechanical injury to the bowel wall which could result in perforation or excessive bleeding [2]. Ideal submucosal injection solutions should have the following characteristics: (1) provide a long-lasting and high submucosal cushion, (2) safe and nontoxic, (3) inexpensive, (4) readily available, (5) easy to inject, and (6) preserve specimen tissue for accurate histopathological lesion assessment [3, 4]. In recent years, various submucosal injection solutions such as normal saline, hyaluronic acid, glycerol, dextrose water, fibrinogen mixture, hydroxypropyl methylcellulose, and even autologous blood have been used in the EMR procedure [5, 6].

We present 3 cases where a lifting agent was employed in the EMR procedure, and subsequent histological analysis with hematoxylin and eosin-stained slides revealed submucosal amorphous deposits that were initially thought to represent amyloid, mucin, or granulomatous inflammation. Due to these differential diagnoses and concerns for the presence of potential mucinous malignancy or a separate accompanying pathological process, additional stains were performed on the tissue sections including mucicarmine, Congo red as well as stains for organisms, and pankeratin immunohistochemical stains to exclude the presence of nests of epithelial cells within the mucin-like pools that were identified in one case.

## 2. Case Reports

### 2.1. Case 1

**Figure 1:**
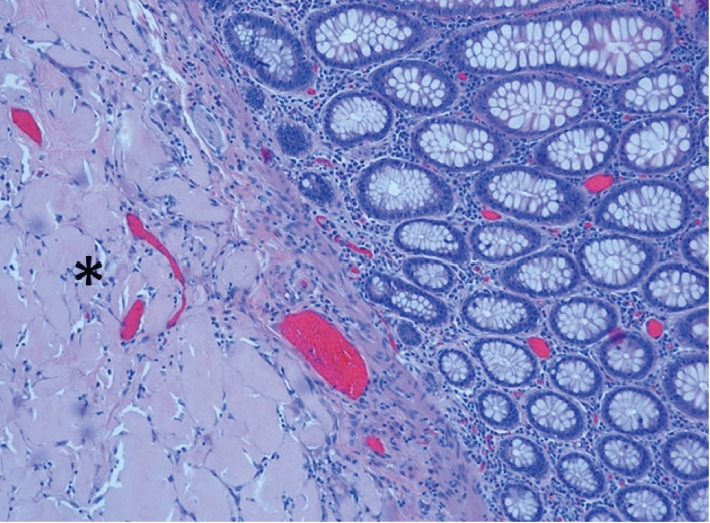
Amorphous material resembling amyloid (∗) in submucosal tissue. H and E stain ×10.

**Figure 2:**
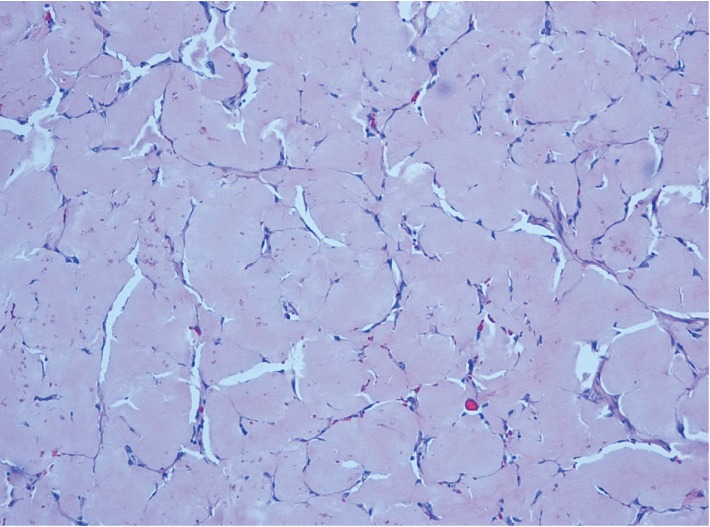
Amorphous eosinophilic material resembling amyloid effacing submucosal connective tissue. H and E stain ×20.

The patient was a 73-year-old male that underwent a laparoscopic-assisted hemicolectomy for a large sessile polyp identified in the ascending colon. Evaluation of the resection specimen demonstrated the presence of a large sessile adenomatous polyp with focal high-grade dysplasia. In the resection specimen, the submucosal tissue was expanded by dense eosinophilic material with a waxy appearance that resembled amyloid (Figures 1 and 2). No specific annotation was present on the surgical pathology request form related to any previous manipulation of the lesion, and additional stains were done to evaluate the eosinophilic deposits noted in this material from the resection specimen. The eosinophilic material was negative for Congo red and mucicarmine staining. Subsequent discussion with the surgeon indicated that, several weeks previously, an EMR had been attempted with the use of a lifting agent marketed as ORISE™. However, technical issues experienced during the EMR had resulted in the procedure being terminated prior to completion of the submucosal resection, and the patient had been referred to surgery for laparoscopic partial colectomy.

### 2.2. Case 2

**Figure 3:**
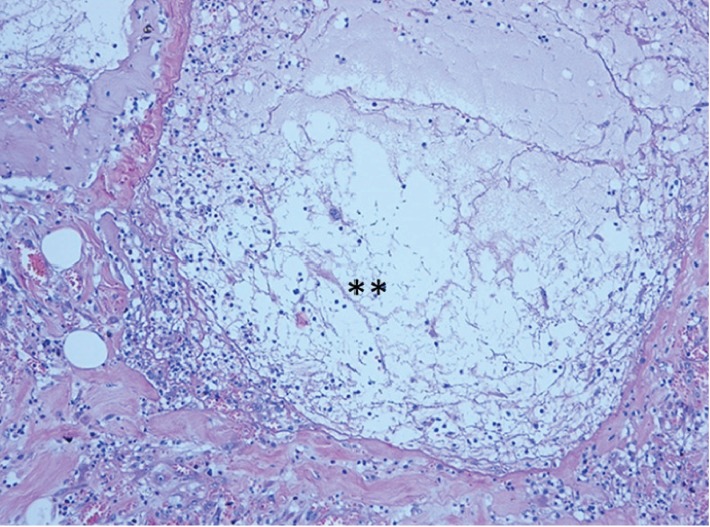
Gel with mucin-like appearance (∗∗) and associated inflammation present in expanded submucosa. H and E stain ×20.

**Figure 4:**
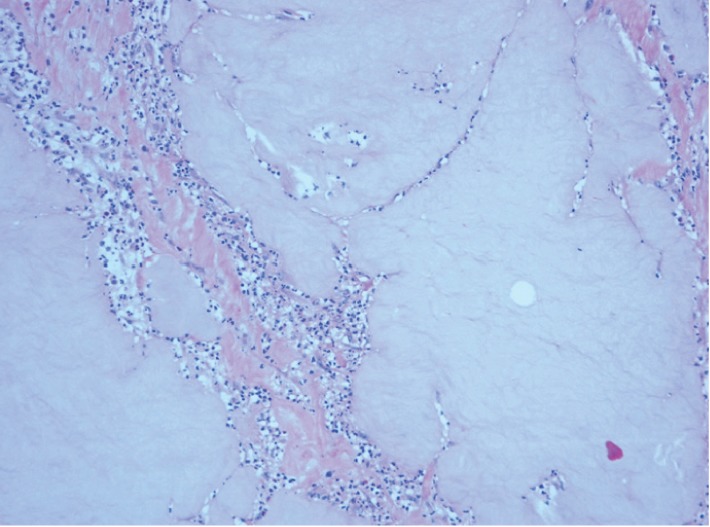
Gel demonstrating features typical of ORISE™ with associated inflammation identified in additional sections. H and E stain ×20.

The patient was a 60-year-old male that presented with a large sessile adenomatous polyp present in the ascending colon. An EMR was undertaken. On examination of the initial EMR specimen, a focus of invasive carcinoma was identified in association with a tubulovillous adenoma exhibiting high-grade dysplasia. Lymphovascular invasion was noted in the EMR specimen, and the patient was subsequently referred for a laparoscopic hemicolectomy. Evaluation of the resected segment of bowel demonstrated an area of ulceration related to the initiating EMR procedure along with pools of intensely inflamed pale eosinophilic debris that resembled mucin (Figure 3). As the initial EMR was attempted at an outside facility, no history was available related to the use of an injectable submucosa lifting agent. Additional studies were undertaken to evaluate this specimen for the presence of epithelial cell aggregates within the inflamed mucin-like debris. However, no evidence of residual or metastatic tumor was identified, and after additional tissue sections were submitted, aggregates of dense eosinophilic material resembling submucosa lifting agent were identified at the periphery of the initial EMR site (Figure 4). Subsequent discussion with the referring gastroenterologist confirmed that ORISE™ had been used in the original EMR procedure.

### 2.3. Case 3

**Figure 5:**
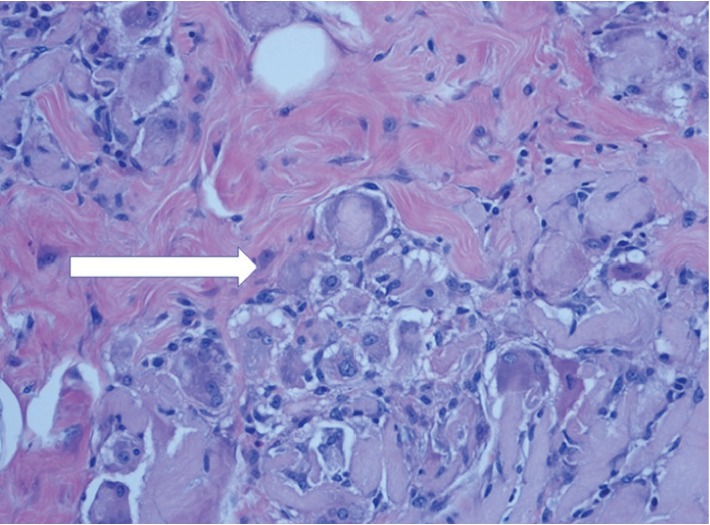
Gel with associated giant cell granulomatous response (arrow). H and E stain ×40.

**Figure 6:**
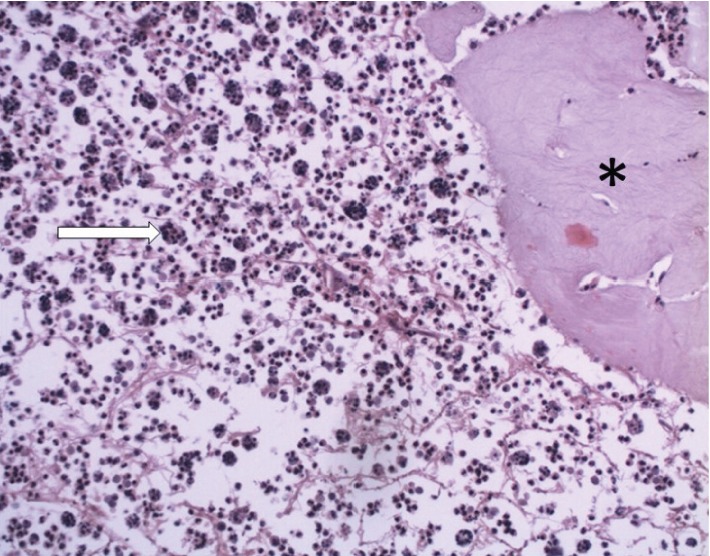
Neutrophil-laden macrophages (arrow) in inflammatory response associated with injected gel (∗). H and E stain ×20.

The patient was a 58-year-old female that had undergone an attempt at an EMR procedure at an outside medical facility. The EMR procedure had been terminated prior to completion of the resection, and the patient was then referred for laparoscopic hemicolectomy. The hemicolectomy specimen demonstrated the presence of a large sessile tubulovillous adenoma with a small focus of mucosal ulceration. No invasive carcinoma was identified in the resection specimen, but sections demonstrated the presence of aggregated multinucleate-type giant cells showing features suggestive of granulomatous inflammation (Figure 5) and long with aggregates of mucinous material showing aggregates of neutrophil-laden macrophages (Figure 6). Special stains for organisms were negative, and additional sections showed aggregates of dense eosinophilic material resembling the gel-like mucosal lifting agent as previously seen in cases 1 and 2.

## 3. Discussion

EMR has now become a standard procedure for the management of noninvasive mucosal tumors and adenomas of the gastrointestinal tract. In the EMR procedure, the removal of these lesions is facilitated by the use of injectable submucosa lifting agents which permit improved visualization of the lesion, increased separation of the lesion from the underlying connective tissue, and thus mitigate against perforation and bleeding during the EMR procedure. A number of fluids have been tried as a submucosal lifting agent in the EMR procedure, and recently, a submucosal injectable gel marketed as ORISE™ (Boston Scientific, 300 Boston Scientific Way, Marlborough, MA 01752-1234, USA) has entered the market. These injectable lifting agents create some degree of artefact which may be mistaken for other pathologies. Unresorbed ORISE™ submucosal injectable gel may present a unique amyloid or mucinous-like appearance on routine hematoxylin and eosin-stained sections, but once recognized, this artifact can be easily recognized thus preventing the use of unnecessary additional studies. This paper highlights the pathology findings associated with submucosal injection of a lifting gel as part of EMR and illustrates the importance of complete information being provided to the pathology department, particularly when novel technologies are being employed.
